# Supplementary material for: Rotational and dilational reconstruction in transition metal dichalcogenide moiré bilayers
Source: Nat Commun. 2023 May 24;14:2989. doi: 10.1038/s41467-023-38504-7 (PMC10209090; doi:10.1038/s41467-023-38504-7)
Supplement: Supplementary file 1 — Supplementary Information [file 41467_2023_38504_MOESM1_ESM.pdf]

Supplementary Information for

Rotational and Dilational Reconstruction in Transition Metal Dichalcogenide Moiré Bilayers

Madeline Van Winkle[1,†], Isaac M. Craig[1,2,3,†], Stephen Carr[4,5], Medha Dandu[2], Karen C. Bustillo[2], Jim Ciston[2], Colin Ophus[2], Takashi Taniguchi[6], Kenji Watanabe[7], Archana Raja[2], Sinéad M. Griffin[2,3], and D. Kwabena Bediako[1,8,*]

[1]Department of Chemistry, University of California, Berkeley, CA 94720, USA
[2]Molecular Foundry, Lawrence Berkeley National Laboratory, Berkeley, CA 94720, USA
[3]Materials Sciences Division, Lawrence Berkeley National Laboratory, Berkeley, CA 94720, USA
[4]Department of Physics, Brown University, Providence, RI 02912, USA
[5]Brown Theoretical Physics Center, Brown University, Providence, RI 02912, USA
[6]International Center for Materials Nanoarchitectonics, National Institute for Materials Science, 1-1 Namiki, Tsukuba 305-0044, Japan
[7]Research for Functional Materials, National Institute for Materials Science, 1-1 Namiki, Tsukuba 305-0044, Japan
[8]Chemical Sciences Division, Lawrence Berkeley National Laboratory, Berkeley, CA 94720, USA
[*]Correspondence to: bediako@berkeley.edu
[†]These authors contributed equally to this work

Table of Contents

1. Sample preparation
2. Derivation of the fitting function
3. Displacement fitting procedure
4. Displacement unwrapping procedure
5. Rigid moiré subtraction
6. Twist angle, lattice constant mismatch, and heterostrain calculation
7. Relaxation simulations
8. Classification of local stacking type for statistics
9. Effect of corrugations on volumetric strain
10. Expected moiré lattice orientation for rotation calibration
11. Uncertainty in strain measurement

1 Sample preparation

Moiré homobilayers were prepared using the 'tear-and-stack' technique.[1] Briefly, a polybisphenol-A-carbonate/polydimethylsiloxane (PC/PDMS) stamp was used to pick up thin (5–10 nm) hexagonal boron nitride (hBN) from a SiO_2/Si substrate. The hBN was then used to pick up part of a MoS_2 monolayer (HQ Graphene), also on SiO_2/Si, tearing the monolayer in half. The remaining monolayer half was then rotated by θ_m before being picked up. The entire heterostructure was directly stamped onto a TEM grid from Norcada (200 nm silicon nitride with 2 μm holes for imaging, Supplementary Figs. 1a,b). Low-magnification dark-field TEM images (Supplementary Figs. 1c,d) were collected to identify regions of interest prior to 4D-STEM measurements. Dark-field images were acquired using a Gatan UltraScan camera on a Thermo Fisher Scientific Titan-class microscope operated at 60 kV.

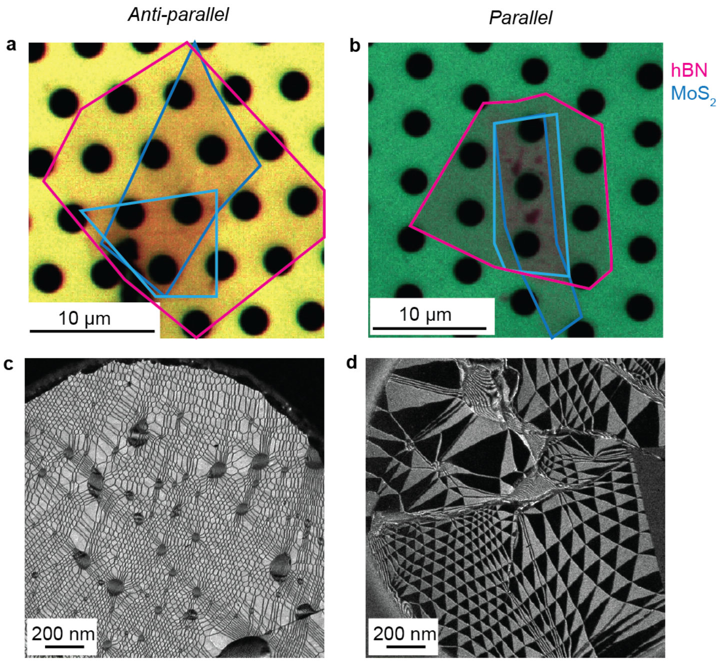

Supplementary Fig. 1. Preliminary characterization of moiré homobilayers. (a,b) Optical micrographs of example anti-parallel- (left) and parallel-stacked (right) hBN/MoS_2/MoS_2 heterostructures on silicon nitride TEM grids. **(c,d)** Corresponding low-magnification dark-field TEM images, collected using $1\bar{2}10$ and $10\bar{1}0$ diffraction peaks, respectively.

Heterobilayers were prepared using a similar transfer technique, but using separate MoS_2 and

$WSe2_2$ (HQ Graphene) monolayers with straight flake edges aligned to target an interlayer twist angle close to 0°(or 60°). For hBN encapsulation studies, two heterobilayer samples were prepared, each of which contained three regions – one with top and bottom hBN, one with top hBN, and one with no hBN – to ensure that the twist angle was approximately constant over the three areas. The crystallographic orientation (parallel vs anti-parallel) of stacked MoS_2 and WSe_2 monolayers cannot be pre-determined during fabrication or distinguished from optical micrographs or dark-field TEM images (Supplementary Figs. 2a–h) and was therefore measured using polarization-resolved second harmonic generation (SHG) spectroscopy (Supplementary Figs. 2i,j). By comparing the relative SHG intensities in the bilayer and monolayer regions, we identified whether there was a parallel (near 0°) or anti-parallel (near 60°) orientation between the MoS_2 and WSe_2. In the case of parallel (anti-parallel) stacking, SHG intensity in the bilayer is greater than (less than) the sum of intensities from the two monolayers.[2]

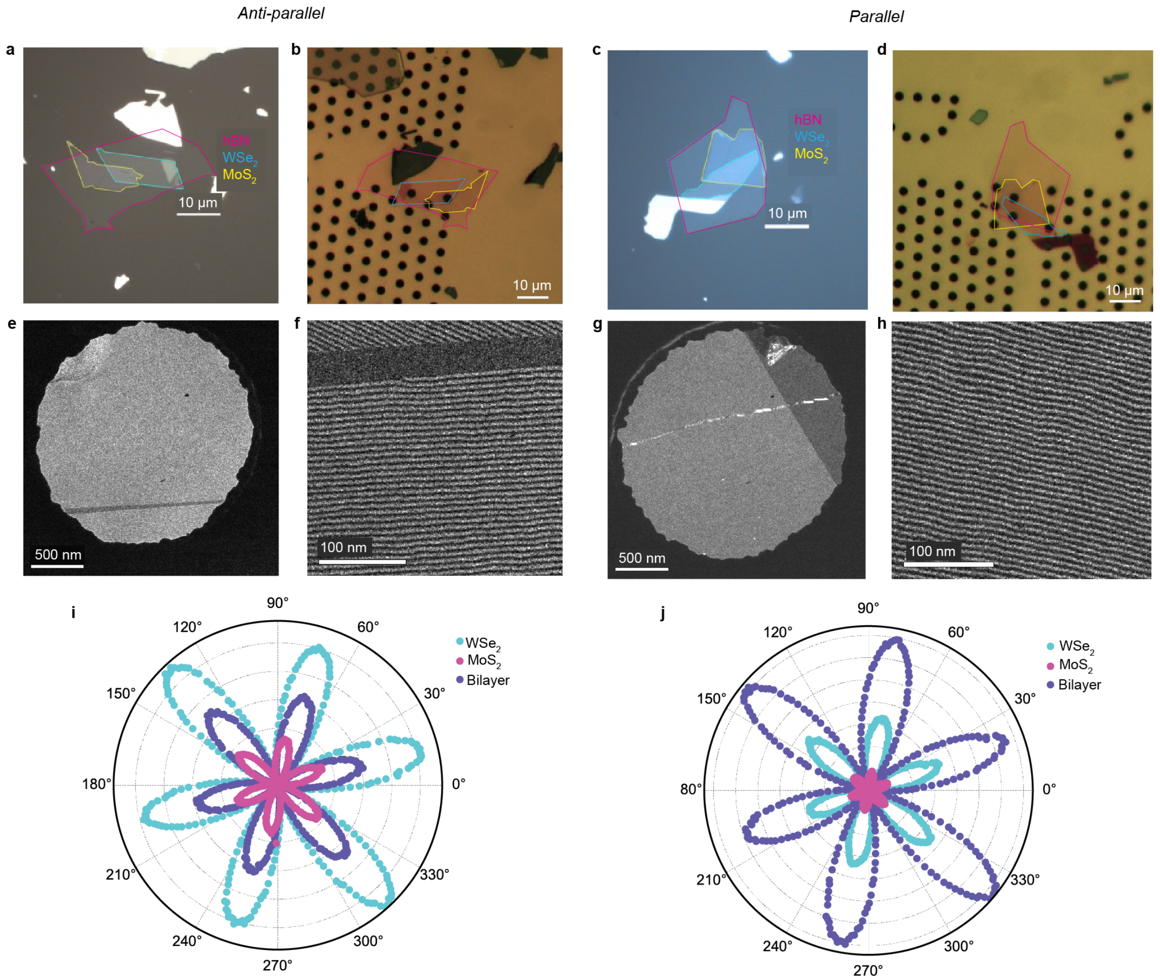

Supplementary Fig. 2. Preliminary characterization of moiré heterobilayers. Optical micrographs of anti-parallel- (left) and parallel-stacked (right) hBN/WSe_2/MoS_2 heterostructures **(a,c)** on PC/PDMS stamps and **(b,d)** on silicon nitride TEM grids. Corresponding low-magnification dark-field TEM images and polarization-resolved SHG measurements are shown in **(e–h)** and **(i,j)**, respectively. Dark-field TEM images were collected using the $10\bar{1}0$ diffraction peak.

2 Derivation of the fitting function

In order to arrive at our expression for modulation of the Bragg disk overlap intensity as a function of interlayer displacement, we first assume that we have a generic bilayer structure in which each of the two layers is represented by a projected electrostatic potential $V_1(\mathbf{r})$ and $V_2(\mathbf{r})$. We denote the interlayer displacement between the atomic coordinates of the two layers in the xy plane as $\mathbf{u} = (u_x, u_y)$ and assume the gap between the layers is negligible such that the outgoing electron beam wavefunction is well-described by a phase shift due to the total electrostatic potential $V_1(\mathbf{r}) + V_2(\mathbf{r})$ (*i.e.* we are assuming that diffraction takes place within a single plane). Accounting for the offset between layers, the total projected potential is then given by the sum $V_1(\mathbf{r} - \mathbf{u}/2) + V_2(\mathbf{r} + \mathbf{u}/2)$. This convention is consistent with our choice to work in the coordinate system defined by the average of the two monolayer lattices. Using the weak phase object approximation, the ψ associated with the outgoing electron beam can therefore be expressed as the following, in which ψ_0 represents the wavefunction of the unscattered electron beam and σ is the relativistic interaction parameter.

$$\psi(\mathbf{r}) \approx (1 + i\sigma V_1(\mathbf{r} - \mathbf{u}/2) + i\sigma V_2(\mathbf{r} + \mathbf{u}/2))\psi_0(\mathbf{r}) \tag{1}$$

Here, the use of the weak phase object approximation is well motivated by the sample thickness being much less than the depth of field of the electron probe.[3,4] In the analysis that follows, we will also assume a fully focused probe such that the contrast transfer function (and thus the more complex interference fringes encountered in holographic approaches [5,6]) does not appear.

The Fourier space intensity (*i.e.* the measured intensity in the region of Bragg disk overlap) $I(\mathbf{k})$ is then given by the following, in which $\circledast$ denotes convolution.

$$I(\mathbf{k}) = |\psi(\mathbf{k})|^2 = |(\delta(\mathbf{k}) + i\sigma \sum_{\mathbf{g}} \delta(\mathbf{k} - \mathbf{g})(V_1(\mathbf{g})e^{i\pi \mathbf{g}\cdot\mathbf{u}} + V_2(\mathbf{g})e^{-i\pi \mathbf{g}\cdot\mathbf{u}})) \circledast \psi_0(\mathbf{r})|^2 \tag{2}$$

Assuming that the convergence semi-angle is chosen to ensure that Bragg disks within a

single layer do not overlap, we arrive at the following expression.

$$I(\mathbf{k}) = |\psi_0(\mathbf{k})|^2 + \sigma^2 \sum_{\mathbf{g}} |\psi_0(\mathbf{k}-\mathbf{g})|^2 (|V_1(\mathbf{g})|^2 + |V_2(\mathbf{g})|^2 + 2Re(V_1(\mathbf{g})V_2^*(\mathbf{g})e^{2i\pi\mathbf{g}\cdot\mathbf{u}})) \quad (3)$$

This is equivalent to the following, in which we defined $A(\mathbf{g}) = 4\,\mathrm{Re}(V_1(\mathbf{g})V_2^*(\mathbf{g}))$, $B(\mathbf{g}) = 4\,\mathrm{Im}(V_1(\mathbf{g})V_2^*(\mathbf{g}))$, and $C(\mathbf{g}) = (|V_1(\mathbf{g})|^2 + |V_2(\mathbf{g})|^2 - 2\,\mathrm{Re}(V_1(\mathbf{g})V_2^*(\mathbf{g})))$.

$$I(\mathbf{k}) = |\psi_0(\mathbf{k})|^2 + \sigma^2 \sum_{\mathbf{g}} |\psi_0(\mathbf{k}-\mathbf{g})|^2 (A(\mathbf{g})\cos^2(\pi\mathbf{g}\cdot\mathbf{u}) + B(\mathbf{g})\sin(\pi\mathbf{g}\cdot\mathbf{u})\cos(\pi\mathbf{g}\cdot\mathbf{u}) + C(\mathbf{g})) \quad (4)$$

At each real space coordinate $\mathbf{r}$, we therefore have the following relationship between the projected displacement vector $\mathbf{u}$ and the modulation in intensity I_j for a set of selected Bragg disks at positions $\mathbf{g}_j$, where $A_j = A(\mathbf{g}_j)$, $B_j = B(\mathbf{g}_j)$, and $C_j = C(\mathbf{g}_j)$ are the coefficients that we treat as fitting parameters which may in general be different for each region of overlap.

$$I_j = A_j \cos^2(\pi\mathbf{g}_j\cdot\mathbf{u}) + B_j \cos(\pi\mathbf{g}_j\cdot\mathbf{u})\sin(\pi\mathbf{g}_j\cdot\mathbf{u}) + C_j \quad (5)$$

We note that when the projected real space potentials are symmetric with respect to inversion (in the plane perpendicular to the electron beam), it is straightforward to show that the Fourier space potentials are necessarily purely real. As a consequence, $C_j = 0$ in materials whose in-plane projections preserve inversion symmetry. Further, B_j vanishes in homobilayer structures when $V_1 = V_2$, resulting in an intensity modulation proportional to $\cos^2(\pi\mathbf{g}\cdot\mathbf{u})$ in accordance with previous work.[4]

3 Displacement fitting procedure

We used a least squares fitting procedure to obtain the optimal interlayer displacements $\mathbf{u}$ and coefficients A_j, B_j, C_j. Throughout our analysis we used the overlap regions in the twelve first-order Bragg disks and assumed that the coefficients remained constant over the sample field of view, resulting in 36 total coefficient variables and $n_x n_y$ displacement variables for a n_x by n_y scan. Fitting the displacements and coefficients in tandem allowed us to both parallelize the procedure and to use a linear optimization for the coefficients.

First, we normalized I_j, assumed $A_j = 1, B_j = 0, C_j = 0$, and determined the optimal displacement vector independently at each pixel using a quasi-Newton non-linear least squares optimization (specifically the trust region reflective algorithm as implemented in scipy).[7] We used a uniform grid of 9 inital guesses for $\mathbf{u}$ to decrease the chance of obtaining local minima. The values of $\mathbf{u}$ were constrained to reside within a single unit cell such that $\mathbf{u} = c_1\mathbf{a}_1 + c_2\mathbf{a}_2$ with $|c_1| \leq 1/2, |c_2| \leq 1/2$, where $\mathbf{a}_1, \mathbf{a}_2$ are the average of the monolayer real space lattice vectors rotated into a convenient basis (see Methods section on rotation calibration). We subsequently determined the optimal coefficients A_j, B_j, C_j given this $\mathbf{u}$ independently for each of the twelve Bragg disk intensities I_j using linear least squares. These two steps were then repeated, where subsequent iterations used the previous iteration $\mathbf{u}$ in the pixel of interest and all four directly adjacent pixels as starting conditions instead of the uniform grid. We found that the $\mathbf{u}$ values converged within 5 iterations of this procedure. To avoid over-fitting and for computational speed, we found it effective to first bin the displacement field (using an L_2 norm and a bin width of 2) and perform the aforementioned procedure to obtain the coefficients and $\mathbf{u}_{bin}$. The raw $\mathbf{u}$ were then fit using $\mathbf{u}_{bin}$ as starting conditions and the coefficients deemed optimal for the binned data, which we held fixed. The final $\mathbf{u}$ were converted from dimensionless units assuming lattice constants a_0 of 0.315 and 0.328 nm for MoS_2 and WSe_2 (values from HQ Graphene) respectively as the data acquisition was not set up to ensure an unbiased estimate of these values. This procedure is summarized in Supplementary Fig. 4a.

4 Displacement unwrapping procedure

The displacement unwrapping procedure amounts to finding the optimal (n,m,s) at each pixel location given $\mathbf{u}_{fit}$ such that the Euclidean distance of $\mathbf{u}_{unwrap}$ from its neighbors is minimized. This yields a continuously varying $\mathbf{u}_{unwrap}$ amenable to differentiation. While it is in principle possible to circumvent such a procedure through accounting for the degeneracy of $\mathbf{u}$ in the differentiation process, we found approaches along these lines less robust to noise due to the decreased ability to smooth the data prior to differentiation.

$$\mathbf{u}_{unwrap} = s\mathbf{u}_{fit} + n\mathbf{a}_1 + m\mathbf{a}_2 \qquad n, m \in Z, s = \pm 1 \tag{6}$$

Instead, we chose to first partition the data into zones expected to have the same offsets n and m. For most of the data sets we used the Watershed segmentation algorithm[8] on $|\mathbf{u}|$, which proved effective in unwrapping displacement fields with decently large regions of $\mathbf{u} \approx 0$ stacking separated by thin boundaries (all but the parallel stacked homobilayers). For the parallel stacked homobilayers, which contained small $\mathbf{u} \approx 0$ stacking regions, a Voronoi partition[7] instead proved more robust to noise. After segmentation, the zone offsets (n,m) of each region were determined based on the region's connectivity, as shown in Supplementary Fig. 3. For instance, in our convention, regions connected by SP1(SP2) soliton walls have the same $m(n)$ offset and a $n(m)$ offsets differing by ± 1, allowing us to successively assign all zones with a breadth first search (starting from the region closest to the center of the data). Large sample deformations will result in the breakdown of this simple algorithm, although we did not find more elaborate approaches necessary for this study. The s of each pixel was then chosen to maximize the local curl in data sets for which $B_j \approx 0$ introduced a sign ambiguity.

Given these estimates for the displacement orientation and lattice vector offsets, we then used an integer program to optimize the parameters along the zone boundaries. The optimization of (n,m,s) along the zone boundaries was accomplished using the following procedure, which

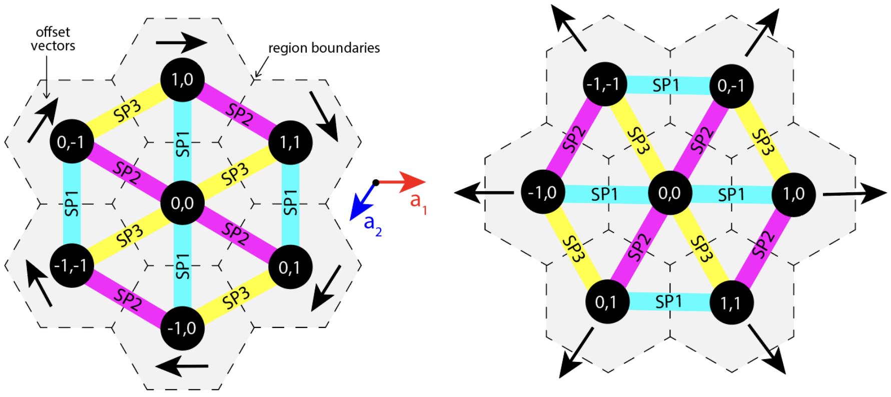

Supplementary Fig. 3. Displacement field partitioning. Region offsets determined geometrically for a twisted homobilayer structure (left) and untwisted heterobilayer structure (right). The integer offsets (n, m) of each zone are shown in the black centers of each hexagonal region, and the corresponding offset vectors $n\mathbf{a}_1 + m\mathbf{a}_2$ are depicted as black arrows. In practice, the heterobilayer displacement fields were first rotated (changing only the lattice vector convention) so that they could be processed similarly to the twisted displacement fields and then rotated back to obtain the diverging displacement field expected for a heterobilayer.

proved sufficient for obtaining smoothly varying displacement fields in this work. Before using a more costly integer program, we first chose the (n,m,s) offsets that gave a $\mathbf{u}_{unwrap}$ closest to the region local mean $\mathbf{u}$ within a 2 x 2 pixel moving window propagated outwards from the center of the data. Both previously assigned pixels and those assigned with confidence in the geometric partition (within 1% of the maximum distance from a region center) were held constant. Each 2 x 2 region after the initial solve was chosen to contain at least one previously assigned pixel. Following this, we identified contours in the data associated with $\mathbf{u}_{unwrap}$ discontinuities where adjacent pixels had a $\mathbf{u}_{unwrap}$ difference larger than what could be obtained by choosing locally optimal (n,m,s). These contours were then used to form a convex mask associated with large regions that had been optimized to a different global offset. For all convex regions of 5 or more pixels, we then used an integer program to successively re-assign the optimal (n,m,s) offsets within these regions, working inwards from their boundaries using a moving 2 x 2 window. This entire process was iterated until the total number of pixels in the convex regions stopped improving. We then used the same integer program to optimize the offsets in a 3 x 3 moving window for areas where

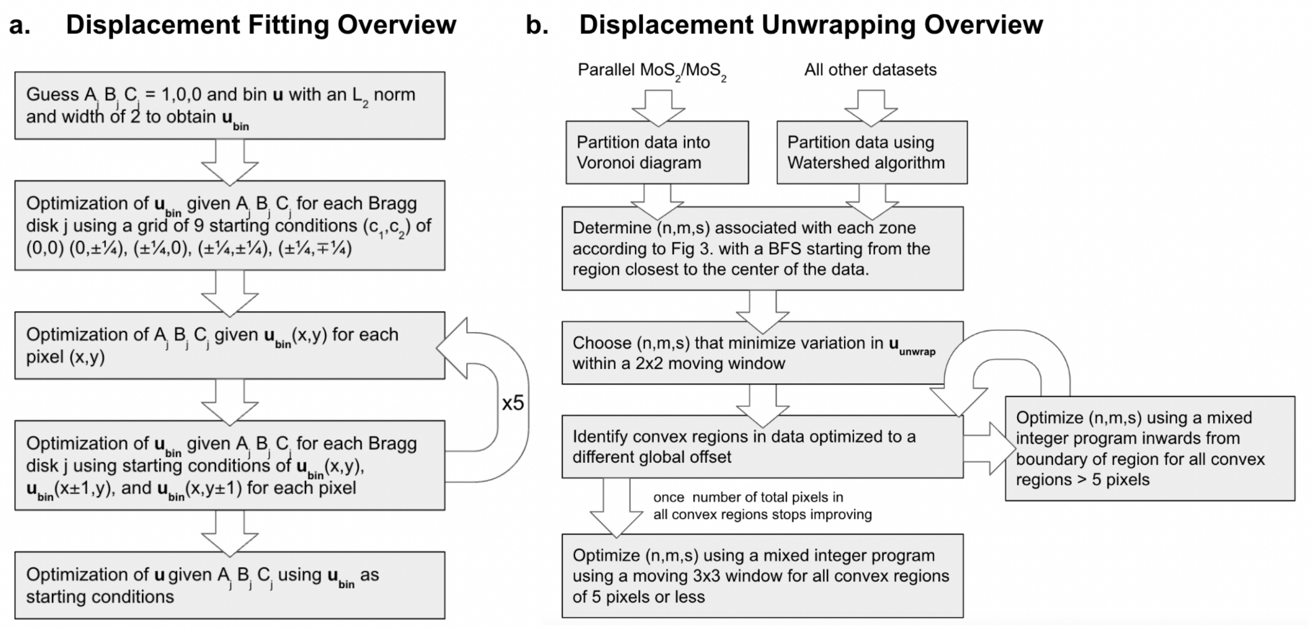

Supplementary Fig. 4. Computational overview. General computational workflow for **(a)** the displacement fitting procedure and **(b)** the unwrapping procedure.

the identified discontinuity regions were 5 pixels or fewer. Throughout, the integer program searched for n and m within ± 2 of the offsets determined from the geometric partitioning. The objective minimized was the sum of all L_2 norms between neighboring pixels, equally weighting both the fixed and variable cells within the region of interest. In practice we optimized s for all datasets (implemented as $s' = 2s - 1$ for convenience in constraints), as we found the sign of $\mathbf{u}$ was more susceptible to experimental noise than its magnitude. The resulting quadratic integer program was solved using APOPT interfaced by GEKKO.[9,10] This procedure is summarized in Supplementary Fig. 4b.

5 Rigid moiré subtraction

In order to assess the local rotation and dilation due to reconstruction, we subtracted off the rotation and dilation expected from a rigid moiré with the same interlayer twist angle, lattice constant mismatch, and/or heterostrain. Assuming an interlayer twist of θ_m and a heterostrain of ϵ in the direction θ_s from the x-axis, we can relate the atomic positions of

the two layers $\mathbf{r}_{ij}^{top}$ and $\mathbf{r}_{ij}^{bottom}$ as follows, where ρ is the material's Poisson ratio.

$$\mathbf{r}_{ij}^{top} = \begin{bmatrix} \cos(\theta_m - \theta_s) & -\sin(\theta_m - \theta_s) \\ \sin(\theta_m - \theta_s) & \cos(\theta_m - \theta_s) \end{bmatrix} \begin{bmatrix} 1+\epsilon & 0 \\ 0 & 1-\rho\epsilon \end{bmatrix} \begin{bmatrix} \cos(\theta_s) & -\sin(\theta_s) \\ \sin(\theta_s) & \cos(\theta_s) \end{bmatrix} \mathbf{r}_{ij}^{bottom}$$

The interlayer displacement of the top layer $\mathbf{u}^{top}$ associated with each atom in the rigid moiré is therefore given by $\mathbf{u}_{ij}^{top} = (\mathbf{r}_{ij}^{top} - \mathbf{r}_{ij}^{bottom})/2$ at each pixel location defined in reference to the bottom layer $\mathbf{r}_{ij}^{bottom} = a_0(x_i, y_j)$ where a_0^{bottom} is the lattice constant of the bottom layer. Computing the local total rotation and dilation from this displacement field, in accordance with the strain mapping procedure outlined in the Methods, results in (to first order in θ_m) a θ_T^{top} of $(2 + \epsilon - \rho\epsilon)\theta_m/2$ and a dilation of $(\epsilon - \rho\epsilon)/2$, both irrespective of θ_s and given in units of a_0^{bottom}.

Similarly, a twisted heterobilayer with an interlayer twist of θ_m and a lattice constant mismatch of δ results in the following displacements. For heterobilayers, we define $\delta = a_L/a_S - 1$ where a_S and a_L are the smaller and larger lattice constants.

$$\mathbf{r}_{ij}^{top} = \begin{bmatrix} \cos(\theta_m) & -\sin(\theta_m) \\ \sin(\theta_m) & \cos(\theta_m) \end{bmatrix} \begin{bmatrix} 1+\delta & 0 \\ 0 & 1+\delta \end{bmatrix} \mathbf{r}_{ij}^{bottom}$$

This is the same expression as the heterostrained homobilayer with $\rho = -1$, $\epsilon = \delta$, and $\theta_s = 0$ such that the interlayer dilation $\nabla \cdot \mathbf{u}^{top} = \delta$ and total intralayer rotation $\theta_T^{top} = (1+\delta)\theta_m/2$. We therefore see that, in terms of local rotations and dilations, heterostrained samples act like heterobilayers with an effective lattice constant mismatch of $(\epsilon - \rho\epsilon)/2$. For the strain maps presented in Figs. 2–5, these rigid values were subtracted from the obtained local rotations and dilations to obtain the reported interlayer reconstruction rotation and dilation. We note that this analysis (and that of the twist angle and lattice mismatch extraction) is carried out using one of the two layers as a reference configuration rather than their average. This slightly modifies the obtained rigid values (by less than 1%) with negligible impact on the resulting analysis.

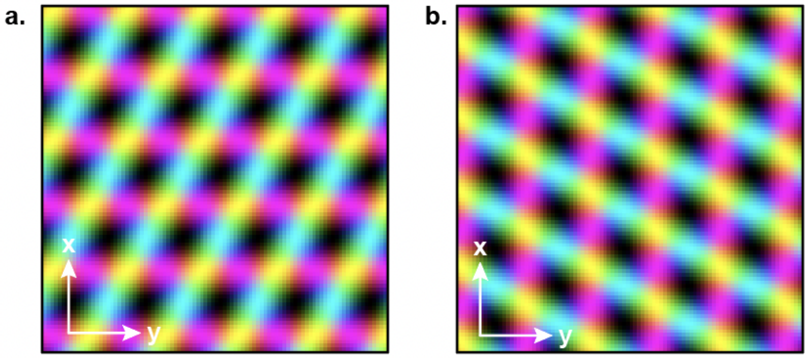

Supplementary Fig. 5. Rigid moiré displacements. Displacement fields for **(a)** rigid twisted homobilayers and **(b)** rigid untwisted heterobilayers, where the lattice vector $\mathbf{a}_1$ is oriented along the x-axis, leading to SP1 solitons oriented along the y-axis and x-axis for the twisted homobilayers and untwisted heterobilayers, respectively.

6 Twist angle, lattice constant mismatch, and heterostrain calculation

Uniaxial heterostrain, local twist, and local lattice constant mismatch were measured through their effects on the moiré pattern. For instance when one of two layers has been twisted by θ_m and subjected to a uniaxial heterostrain ϵ along the direction θ_s from the x-axis, the set of three experimentally accessible real space moire wavelengths λ_i can be expressed in terms of θ_m, θ_s, ϵ, the unstrained monolayer reciprocal lattice vectors $\mathbf{k}_i$, and the Poisson ratio ρ as follows.[11]

$$\lambda_i = \frac{4\pi}{\sqrt{3}} \left| \begin{bmatrix} \cos(\theta_m - \theta_s) & -\sin(\theta_m - \theta_s)) \\ \sin(\theta_m - \theta_s) & \cos(\theta_m - \theta_s) \end{bmatrix} \begin{bmatrix} \frac{1}{1+\epsilon} & 0 \\ 0 & \frac{1}{1-\rho\epsilon} \end{bmatrix} \begin{bmatrix} \cos(\theta_s) & -\sin(\theta_s) \\ \sin(\theta_s) & \cos(\theta_s) \end{bmatrix} \mathbf{k}_i - \mathbf{k}_i \right|^{-1} \tag{7}$$

We used a ρ of 0.23 and 0.25 for P and AP MoS_2 respectively, which were obtained by assuming the material response of each were equivalent to that of the most energetically favorable stacking order (XM or XMMX).[12] While this choice neglects the variation in stacking order, using alternative estimates of ρ has a minor effect on the obtained θ_m and ϵ values.

We obtained the local twist angle and heterostrain for the P and AP homobilayers using Delaunay triangulation[7] on the centers of each MMXX or XMMX region and fitting the resultant moiré wavelengths λ_i to this expression for θ_m, θ_s, ϵ (Eq. 7) with nonlinear least squares. However, as an added complication for heterobilayer systems, the difference in material response in the two layers results in an under-determined set of equations, prohibiting us from relating any asymmetry in the three moiré wavelengths to a well-defined heterostrain for these systems. Further, the average real space moiré wavelength λ in heterobilayer samples is set by both the twist angle and local lattice constant mismatch $\delta = 1 - a_S/a_L$, both of which can vary throughout a given sample.[13] Again, here a_S and a_L are the smaller and larger lattice constants, respectively.

$$\lambda = \frac{(1-\delta)a_L}{\sqrt{\delta^2 + 2(1-\delta)(1-\cos(\theta_m))}} \tag{8}$$

As a result, the local lattice constant mismatch of the heterobilayer samples was then calculated by assuming a constant twist angle for a given data set, which was obtained from the averaged diffraction pattern. To control for heterostrain effects and twist angle heterogeneity, we then redistricted our analysis to heterobilayers samples displaying both minimal asymmetry in the three moiré wavelengths and minimal λ variation.

7 Relaxation simulations

7.1 Density functional theory

In order to model the atomic relaxation vector fields $\mathbf{u}$ of the moiré bilayers via a continuum elasticity model, three pieces of information are necessary. These are the crystal lattice parameter a, the bulk and shear strain moduli K and G, and the stacking-dependent interfacial energy between pairs of layers which is called the generalized stacking fault energy (GSFE) given by $V_{GSFE}(\mathbf{u})$. For all layers' strain moduli, and the V_{GSFE} of MoS_2, we use previously computed values from DFT.[14,15]

For the modeling of the MoS_2/WSe_2 heterointerface, new DFT calculations were needed. We used the Vienna ab initio Simulation package (VASP),[16] and performed slab calculations of the monolayer and heterolayer with a vertical (c) axis of 30 Å to prevent interaction between periodic images. The electronic structure was optimized on a Γ-centered k-grid of size $21 \times 21 \times 1$, the energy cutoff was set to 500 eV, and the energy smearing was set to 50 meV. The meta-GGA functional SCAN+rVV10 [17] was used alongside the PAW-PBE pseudo potentials for all atoms.[18] To obtain the V_{GSFE}, the stacking configurations between the two layers were sampled over a 9×9 grid of the unit-cell. Each of these 36 heterolayer calculations fixed the in-plane location of all atoms, but allowed the vertical positions to relax via a conjugate gradient algorithm. The five lowest harmonic modes of the V_{GSFE} were then extracted, following the formula

$$\begin{aligned} V_{GSFE}(v,w) = \\ & c_0 + c_1(\cos v + \cos w + \cos(v+w)) \\ & + c_2(\cos(v+2w) + \cos(v-w) + \cos(2v+w)) \\ & + c_3(\cos(2v) + \cos(2w) + \cos(2v+2w)) \\ & + c_4(\sin v + \sin w - \sin(v+w)) \\ & + c_5(\sin(2v+w2) - \sin(2v) - \sin(2w)) \end{aligned} \tag{9}$$

which uses normalized stacking-parameters (v, w) which are given by the transformation

$$\begin{pmatrix} v \\ w \end{pmatrix} = 2\pi A_1^{-1} \begin{pmatrix} b_x \\ b_y \end{pmatrix} \tag{10}$$

for $\mathbf{b} = (b_x, b_y)$ the stacking configuration of the top layer and A_1 the matrix composed of the lattice vectors of the bottom layer. For both the P (e.g. near 0° alignment) and AP (e.g. near 60° alignment) of the MoS_2/WSe_2 V_{GSFE}, $\mathbf{b} = 0$ was defined as the highest energy stacking point. For the P configuration, $\mathbf{b} = 0$ corresponds to where both the metals and chalcogenides are vertically aligned. The lowest energy stacking (which occurs when

$v = w = 2\pi/3$) corresponds to where the S and W atoms are vertically aligned. For the AP configuration, $\mathbf{b} = 0$ corresponds to where the S and Se atoms are vertically aligned. The lowest energy stacking (which occurs when $v = w = 4\pi/3$) corresponds to where the Mo and Se atoms (also, W and S atoms) are vertically aligned. The coefficients for the V_{GSFE} are given in Table 2 and those which generate the configuration-dependent equilibrium interlayer distance are given in Table 3.

Material	a	K	G
MoS_2	3.17	49.866	31.548
WSe_2	3.28	43.113	30.770

Table 1: Monolayer parameters extracted from DFT.[14,15] The lattice parameter a is given in units of AA, and the shear moduli K and G are given in units of eV.

Interface	c_0	c_1	c_2	c_3	c_4	c_5
P-MoS_2/MoS_2	27.332	14.020	-2.542	-0.884	0.000	0.000
AP-MoS_2/MoS_2	30.423	12.322	-2.077	-0.783	2.397	0.259
P-MoS_2/WSe_2	32.967	13.888	-3.281	-0.748	-1.139	-0.175
AP-MoS_2/WSe_2	37.233	12.317	-2.691	-0.234	3.535	0.712

Table 2: Coefficients for V_{GSFE} (Eq. 9) for the four interfaces modeled in this work. All values are given in units of meV per unit-cell of MoS_2.

Interface	c_0	c_1	c_2	c_3	c_4	c_5
P-MoS_2/WSe_2	6.561	0.133	0.001	-0.012	-0.001	-0.002
AP-MoS_2/WSe_2	6.558	0.115	-0.002	-0.019	-0.011	-0.013

Table 3: Coefficients for the configuration-dependence of the interlayer distance between metal atoms (using same form as Eq. 9) for the two heterointerfaces, in units of Å.

7.2 Continuum elasticity model

The atomic relaxation of two layers is assumed to be smooth and periodic on the superlattice of the given moiré pattern. It is modeled by two vector fields, $\mathbf{u}_1(\mathbf{r})$ for the bottom layer and $\mathbf{u}_2(\mathbf{r})$ for the top layer, and where $\mathbf{r}$ is the location in the moiré pattern. Note that one can also perform this two-layer relaxation problem in the configuration basis, e.g. replace $\mathbf{r}$ with a pre-relaxation stacking reference $\mathbf{b}_0$, which is the relative stacking configuration of layer 2 relative to layer 1 before any atomic relaxations occur ($u_l = 0$). For systems with only one unique moiré interface, these two approaches are identical, and are related by the

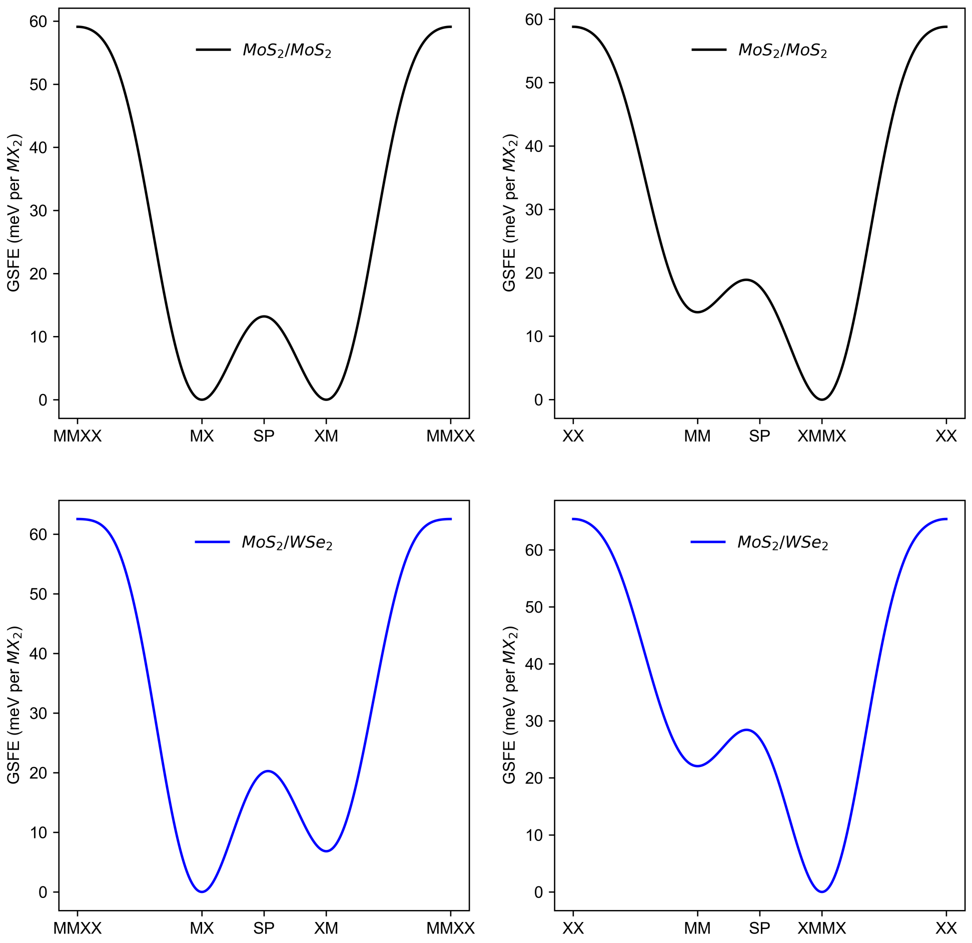

Supplementary Fig. 6. Generalized stacking fault energies. V_{GSFE} as given in Table 2 plotted as a function of a normalized stacking parameter $v = w$. High symmetry stacking configurations are labeled.

linear map which transforms $\mathbf{r}$ to $\mathbf{b}_0$:

$$\mathbf{b}_0(\mathbf{r}) = A_1 A_{sc}^{-1}\mathbf{r} \tag{11}$$

where A_1 is the 2×2 matrix consisting of the unit-cell lattice vectors of the bottom layer, and A_{sc} are the lattice vectors of the moiré superlattice.

In fact, the optimization problem for a given collection of twist angles and heterostrain values is completely specified by just the moiré superlattice A_{sc} (and its angle relative to the unit-cell A_1). For clarity, we define the A_1 unit cell as

$$A_1 = a_1 \begin{pmatrix} \sqrt{3}/2 & \sqrt{3}/2 \\ -1/2 & 1/2 \end{pmatrix} \tag{12}$$

where the primitive lattice vectors are given by the columns of A_1. For a unit cell A_2 defined relative to A_1 by a isotropic heterostrain (e.g. lattice constant difference) of α, a counter-clockwise twist of θ, and a shear heterostrain of magnitude β at an angle ϕ from the A_1 unit-cell, A_{sc} is given by

$$\begin{aligned} A_{sc} &= (SR - I)^{-1} A_1, \\ R &= \begin{pmatrix} \cos\theta & \sin\theta \\ -\sin\theta & \cos\theta \end{pmatrix}, \\ S &= \begin{pmatrix} 1+\alpha+\beta_1 & \beta_2 \\ \beta_2 & 1+\alpha-\beta_1 \end{pmatrix}, \\ \beta_1 &= (\beta/2)\cos(2\phi + \pi/6 - \theta/2), \\ \beta_2 &= (\beta/2)\sin(2\phi + \pi/6 - \theta/2), \end{aligned} \tag{13}$$

with I the 2×2 identity matrix.

For the MoS_2 bilayers, we impose a relaxation symmetry assumption that $\mathbf{u}_2(\mathbf{r}) = -\mathbf{u}_1(\mathbf{r})$. That is to say, the relaxation of the two layers is equal and opposite at a given configuration,

which ensures the effective change in the stacking, $\Delta\mathbf{u} = \mathbf{u}_2 - \mathbf{u}_1$, is evenly split between the two layers to minimize the strain energy. For the MoS_2/WSe_2 heterobilayer, we minimize both $\mathbf{u}_1$ and $\mathbf{u}_2$ independently, as the two layers do not have the same strain moduli.

The total energy is composed of two parts,[14] the elastic strain of each layer and the interfacial energy between them:

$$\begin{aligned}
E &= E_{\text{strain}} + E_{\text{GSFE}}, \\
E_{\text{strain}} &= \sum_{l=1}^{2} \int d\mathbf{r} \frac{1}{2} \mathcal{E}(\nabla \mathbf{u}_l(\mathbf{r})) C_l \mathcal{E}(\nabla \mathbf{u}_l(\mathbf{r})), \\
E_{\text{GSFE}} &= \int d\mathbf{r} V_{GSFE} \left(\mathbf{b}_0(\mathbf{r}) + \mathbf{u}_2(\mathbf{r}) - \mathbf{u}_1(\mathbf{r}) \right).
\end{aligned} \tag{14}$$

with C_l the rank-4 stiffness tensor (consisting of K_l and G_l), and $\mathcal{E}(M) = (M + M^T)/2$. This energy functional and its analytic gradient are implemented as functions which act on an $N \times N$ grid-sampling $\mathbf{u}_l$ over A_{sc}, with $N = 32$. The $\mathbf{u}_l$ are then optimized via the Julia OPTIM package,[19] using the quasi-Newton solver L-BFGS.

Results for the MoS_2 moiré bilayer relaxations are shown in Supplementary Figs. 7–9. The reconstruction rotation maps and shear strain fields in Supplementary Fig 7. align well with the experimental maps provided in Fig. 2 in the main text. Supplementary Figs. 8 and 9 demonstrate the effects of introducing a large heterostrain for P and AP moiré homobilayers, respectively. Similar to the data provided in Fig. 5, we observe that local rotations still dominate the reconstruction process when heterostrain is applied. Qualitative differences between the shear strain distributions in the simulated and experimental data can be attributed to differences in the direction of the applied heterostrain; in the simulations, heterostrain was applied at precisely 90°(Supplementary Fig. 8b,e,h and 9b,e,h) or 0°(Supplementary Fig. 8c,f,i and 9c,f,i) relative to the moiré unit cell, whereas the experimental data sets have a heterostrain angle that deviates from these values.

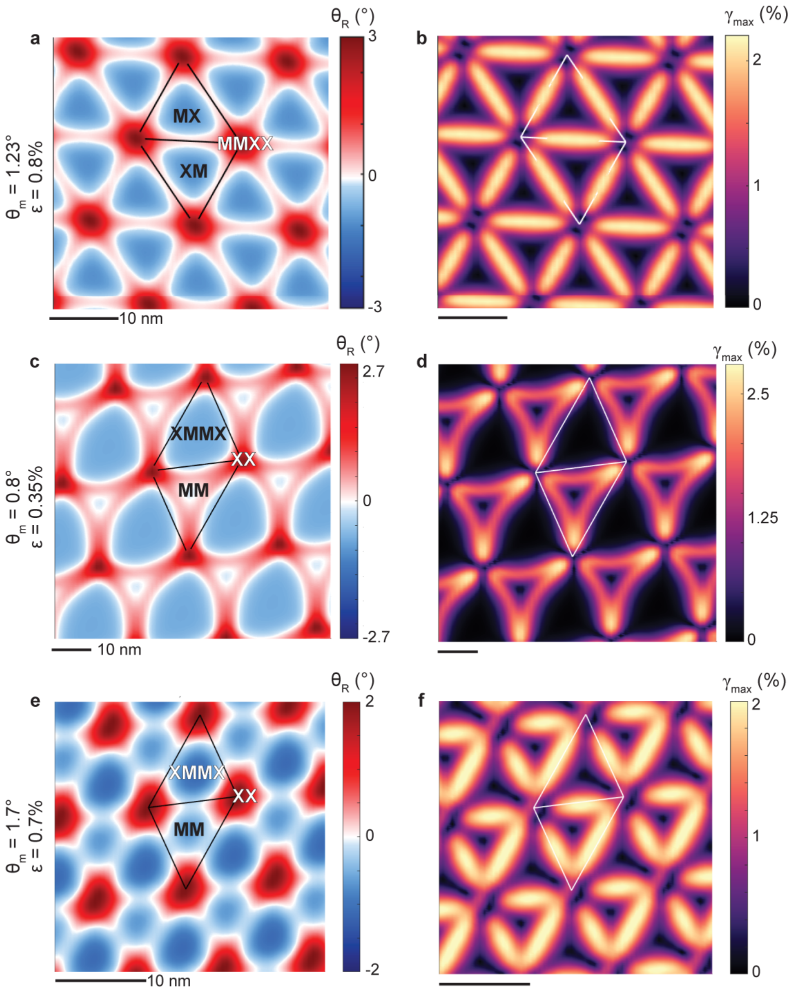

Supplementary Fig. 7. Simulated moiré homobilayer relaxation. Simulated maps of local reconstruction rotation (θ_R) and shear strain (γ_{max}) for **(a,b)** P and **(c–f)** AP MoS_2 moiré homobilayers. θ_m and ϵ indicate the moiré twist angle and heterostrain magnitude, respectively.

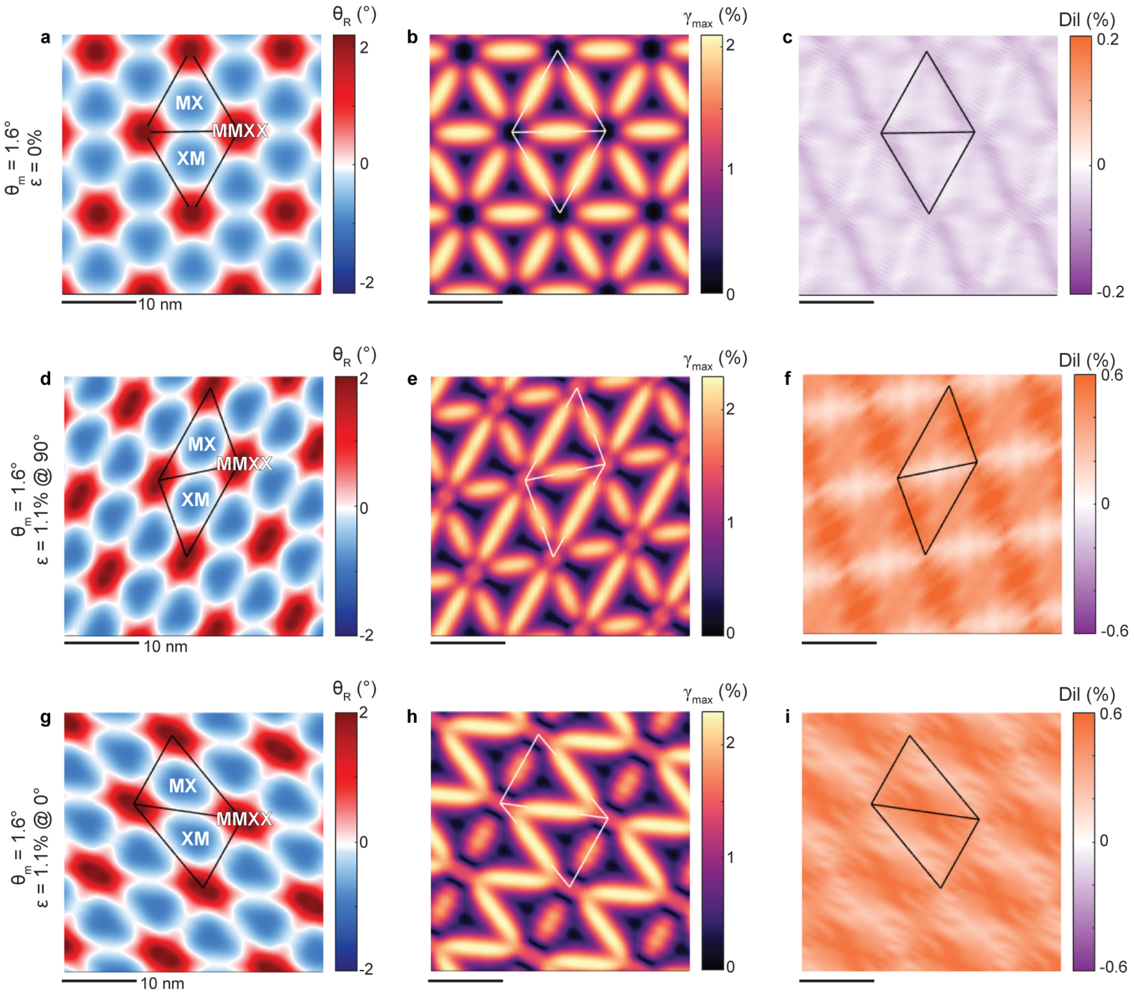

Supplementary Fig. 8. Heterostrain simulations for P moiré homobilayer. Simulated maps of local reconstruction rotation (θ_R), shear strain (γ_{max}), and dilation (Dil) for P MoS_2 moiré homobilayers with varying amounts and directions of applied uniaxial heterostrain. θ_m and ϵ indicate the moiré twist angle and heterostrain magnitude, respectively. Maps are shown for cases of **(a–c)** no heterostrain, **(d–f)** heterostrain applied at 90° relative to the moiré, and **(g–i)** heterostrain applied at 0° relative to the moiré.

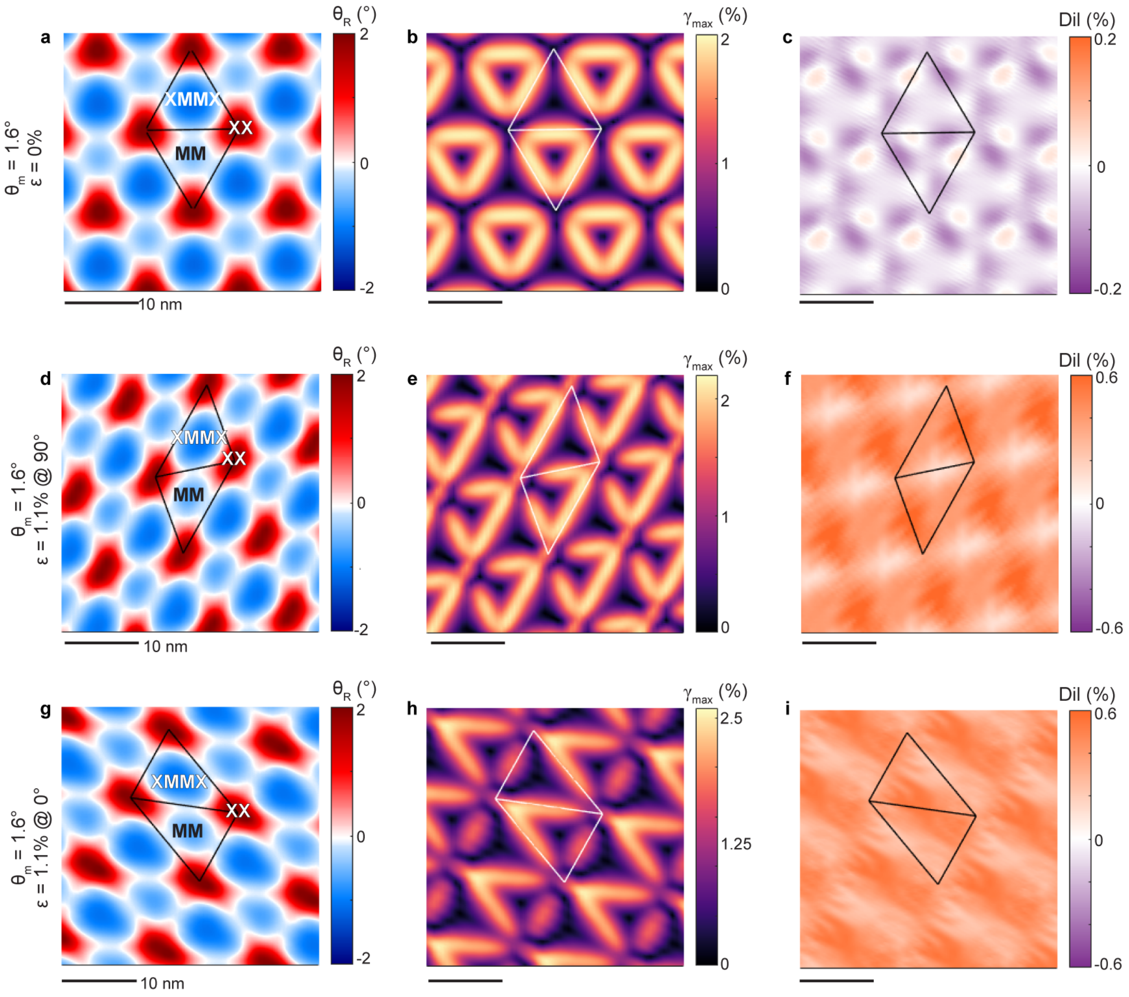

Supplementary Fig. 9. Heterostrain simulations for AP moiré homobilayer. Simulated maps of local reconstruction rotation (θ_R), shear strain (γ_{max}), and dilation (Dil) for AP MoS_2 moiré homobilayers with varying amounts and directions of applied uniaxial heterostrain. θ_m and ϵ indicate the moiré twist angle and heterostrain magnitude, respectively. Maps are shown for cases of **(a–c)** no heterostrain, **(d–f)** heterostrain applied at 90° relative to the moiré, and **(g–i)** heterostrain applied at 0° relative to the moiré.

Relaxation simulations for the MoS_2/WSe_2 heterobilayers are shown in Supplementary Figs. 10–12. Since we extract values for interlayer displacement with the Bragg interferometry method, the experimental dilation and rotation values in Figs. 3,4 of the main text represent net values for the two TMDs in the heterobilayer. However, in contrast, the simulated results distinguish between the relaxation in layer 1 (MoS_2) and layer 2 (WSe_2) (Supplementary Figs. 10,11). Based on the simulations, we find that the physical deformations from the relaxation process are partitioned nearly equally between the two layers, with relaxation in the WSe_2 layer being only 13% stronger than in the MoS_2 layer. To compare directly between the experimental and simulated results, we calculate the net local dilation and reconstruction rotation as $Dil_{net} = Dil_{layer2} - Dil_{layer1}$ and $\theta_{R,net} = \theta_{R,layer2} - \theta_{R,layer1}$ (Supplementary Fig. 12). Consistent with our measurements, the simulations indicate that a combination of reconstruction dilations and rotations are present when the interlayer twist angle is sufficiently large. The fact that there are periodic reconstruction rotations in the simulated P heterobilayer with a non-zero twist but not in our experimental data (Fig. 3j) may be attributed to the difference in twist angle between the two ($1.2°_{sim}$ vs $0.80°_{exp}$), indicating that the threshold for rotational reconstruction is above 0.80°, or to additional out-of-plane corrugations in the sample, as discussed in the main text.

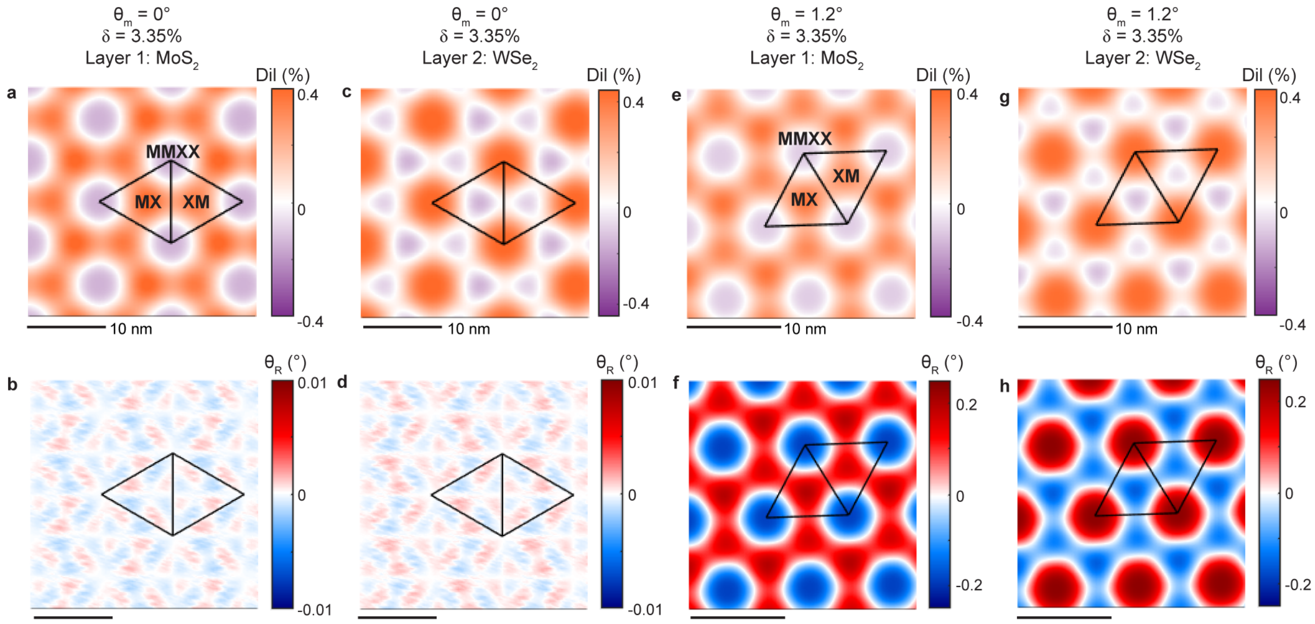

Supplementary Fig. 10. Simulated P heterobilayer relaxation. Simulated maps of local dilation (Dil) and reconstruction rotation (θ_R) within each layer of a P MoS_2/WSe_2 heterobilayer using a lattice constant percent difference (δ) of 3.35% and a moiré twist angle of **(a–d)** 0° and **(e–h)** 1.2°.

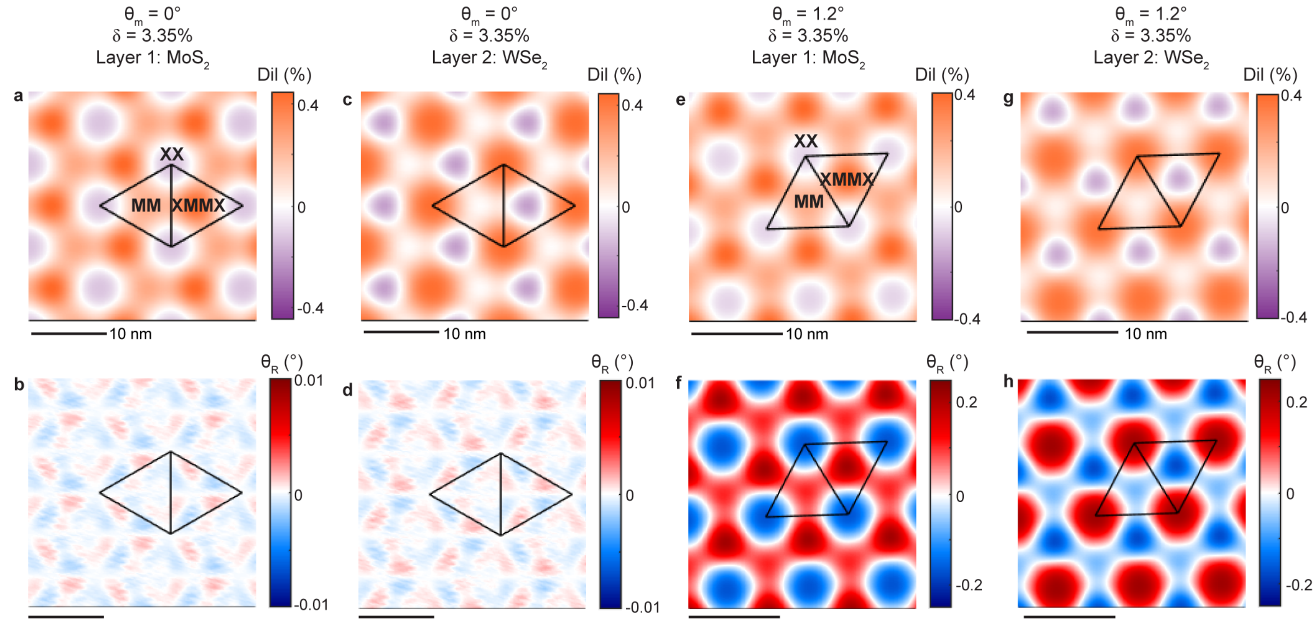

Supplementary Fig. 11. Simulated AP heterobilayer relaxation. Simulated maps of local dilation (Dil) and reconstruction rotation (θ_R) within each layer of an AP MoS_2/WSe_2 heterobilayer using a lattice constant percent difference (δ) of 3.35% and a moiré twist angle of **(a–d)** 0° and **(e–h)** 1.2°.

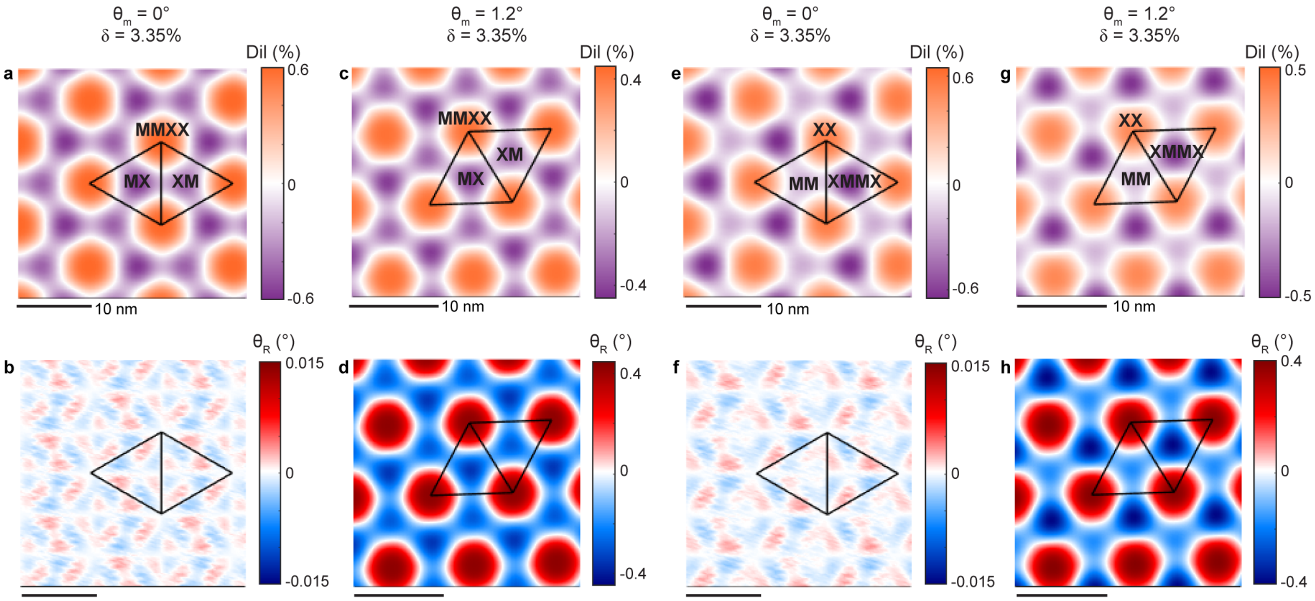

Supplementary Fig. 12. Net relaxation in moiré heterobilayers. Simulated maps of the net local dilation (Dil) and reconstruction rotation (θ_R) in **(a–d)** P and **(e–h)** AP MoS_2/WSe_2 heterobilayers. θ_m and δ indicate the moiré twist angle and lattice constant percent difference, respectively.

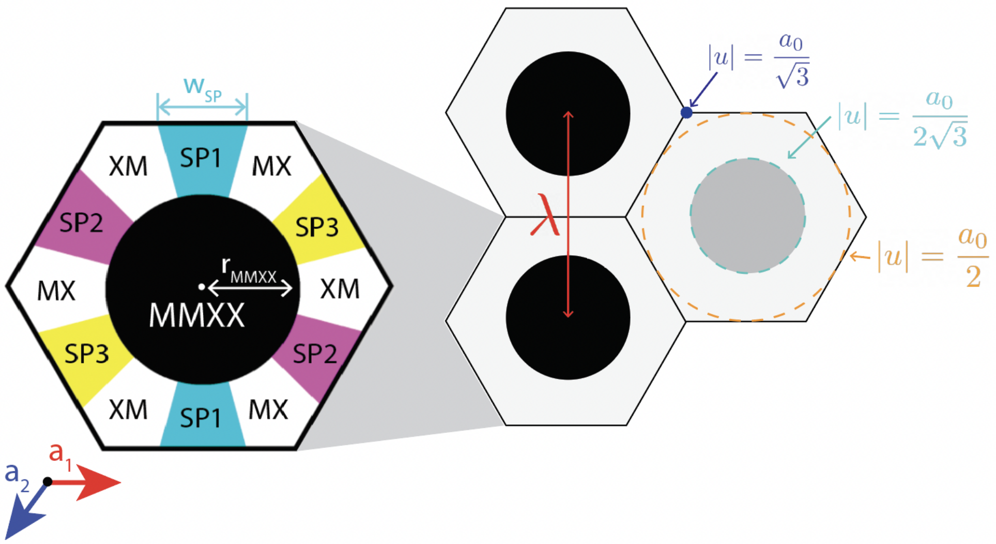

Supplementary Fig. 13. Stacking sequence classification. Schematic depicting classification of stacking type in a rigid twisted bilayer. Displacement vectors with $|\mathbf{u}| < \frac{a_0}{2\sqrt{3}}$, corresponding to the dashed blue contour shown, are assigned MMXX (XMMX) stacking order. We define SP1 regions as having an angular offset from vertical, ϕ, within $\pi/12$ of $(0, \pi, 2\pi)$, SP2 regions as having a ϕ within $\pi/12$ of $(\pi/3, 4\pi/3)$, and so on as shown.

8 Classification of local stacking type for statistics

The displacement vectors were partitioned into stacking order categories to obtain stacking area percentages and statistics as described in the Methods. Defining a_0 as the average lattice constant of the two layers and $\mathbf{u}$ as the interlayer displacement, we classified displacement vectors with $|\mathbf{u}| < \frac{a_0}{2\sqrt{3}}$ as XMMX (MMXX) stacking and the remaining displacements as XX (MX), MM (XM), or SP type stacking according to their angular offset from vertical ϕ, as seen in Supplementary Fig. 13. While the $\mathbf{u}$ associated with XMMX (MMXX) stacking always have $|\mathbf{u}| < \frac{a_0}{2\sqrt{3}}$, corresponding to the contour shown, the distance between these regions depends on the moiré wavelength λ, so that rigid samples are expected to have XMMX (MMXX) stacking radii of $r = \frac{\lambda}{2\sqrt{3}}$ and SP widths of $w_{SP} = \lambda \tan(\pi/12)$. Rigid XMMX (MMXX), XX+MM (XM+MX), and SP percents can be calculated geometrically as 30.2%, 38.5%, and 31.3% respectively.

9 Effect of corrugations on volumetric strain

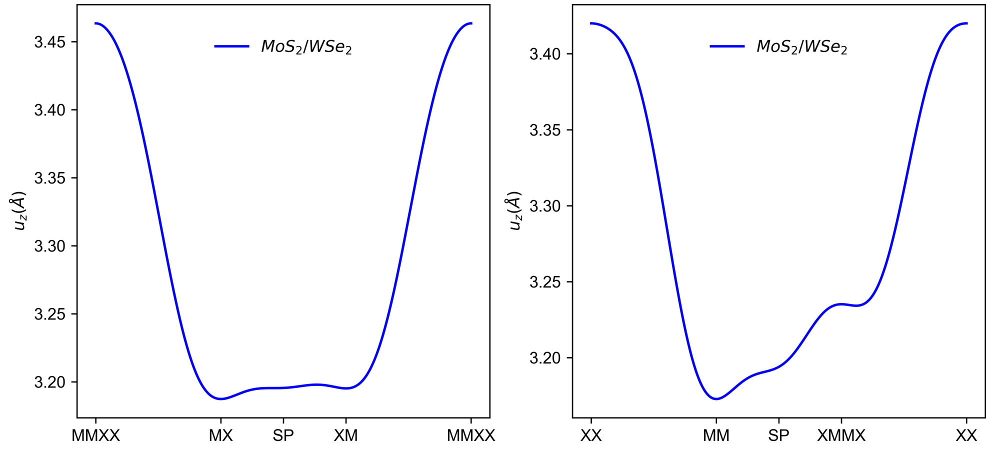

Supplementary Fig. 14. Interlayer spacing variation. Single layer displacement along the z-axis as a function of normalized stacking parameter for a P (left) and AP (right) moiré heterobilayer, excluding any in-plane relaxation.

To predict the relative effect of corrugations on the measured dilations, we use the interlayer distance functionals provided in Ref. [20] for MoS_2 and in Supplementary Note 7.1 for MoS_2/WSe_2. In both cases, we observe that the single layer displacement along the z-axis (u_z^{top}, Supplementary Fig. 14) varies on the order of 0.3Å (resulting in a total interlayer distance of 0.6Å, similar to previously reported values[21,22]) and has extrema at MX and MMXX regions (or MM and XX for AP bilayers). From the MoS_2/WSe_2 interlayer distance functional, we find that the steepest dependence of u_z^{top} on the in-plane displacement is $\partial u_z^{top}/\partial u_x^{top} \approx 0.51$ and occurs when the normalized stacking parameter (see Supplementary Note 7.1) is around 5.3 for P MoS_2/WSe_2. The z-axis displacement will then vary with probe location $\mathbf{r} = (x, y)$ through this dependence on in-plane displacement. We then expand to first order about the location of interest $\mathbf{r}_0$ where $v = 5.3$. Assuming the moiré is controlled only by lattice mismatch such that $\partial u_x^{top}/\partial y = \partial u_y^{top}/\partial x = 0$, this results in the following.

$$u_z^{top}(\mathbf{r}) \approx u_z^{top}(\mathbf{r}_0) + \frac{\partial u_z^{top}}{\partial u_y^{top}} \frac{\partial u_y^{top}}{\partial y} \Delta y + \frac{\partial u_z^{top}}{\partial u_x^{top}} \frac{\partial u_x^{top}}{\partial x} \Delta x \quad (15)$$

Around this location where out of plane displacements are expected to be greatest, local in-plane variations in $\mathbf{u}^{top}$ that correspond to a volumetric strain along the x-axis of $\partial u_x^{top}/\partial x$ will be accompanied by variations in u_z^{top} up to roughly half as large for P MoS_2/WSe_2. We can then define the effective lattice compression along the x-axis between the two layers associated with this change in height such that the intralayer displacement magnitude in the xz plane $|u^{top}|_{xz}$ is related to its in-plane projection u_x^{top} via $|u^{top}|_{xz} = (1 - \delta_x^{cor}/2)u_x^{top}$. An analagous analysis can be carried out to obtain the effective compression along the y-axis δ_y^{cor} expected to be on the same order of magnitude.

$$\delta_x^{cor}/2 = 1 - \left(1 + \left(\frac{\partial u_z^{top}}{\partial u_x^{top}}\right)^2\right)^{-1/2} \tag{16}$$

This corrugation-driven apparent lattice compression will decrease the effective interlayer lattice mismatch, $\delta = 1 - a_S/a_L$ (where a_S and a_L are the smaller and larger lattice constants respectively), by a factor of $1 - (\delta_x^{cor} + \delta_y^{cor})/2$, particularly in regions where the optimal interlayer height is most sensitive to stacking order. These compressions are therefore only expected in the narrow boundaries between XX and XMMX or MM regions for AP bilayers (or between MMXX and MX/XM stacking regions in P bilayers) as seen in Fig. 4k for the AP case. Measured dilations with a magnitude that is greater than the computed values suggest in-plane volumetric deformations in the underlying atomic lattices.

We note that this result is due to the fact that the dilation is measured in the sample with respect to a fixed rigid lattice constant. The corrugation-driven lattice compression will not change the percent lattice mismatch δ when both layers are assumed to deform by the same percent. This is because the corrugation will effectively compress both a_L and a_S by the same factor $(1 - (\delta_x^{cor} + \delta_y^{cor})/2$) leaving the lattice mismatch ($\delta = 1 - a_S/a_L$) and any strain measured relative to $(\delta_x^{cor} + \delta_y^{cor})/2)a_L$ unchanged. However since we measure strain relative to a uniform a_L reference, this effect leads to an apparent decrease in the displacement magnitudes within corrugated regions, manifesting in a smaller divergence and a perceived negative dilation.

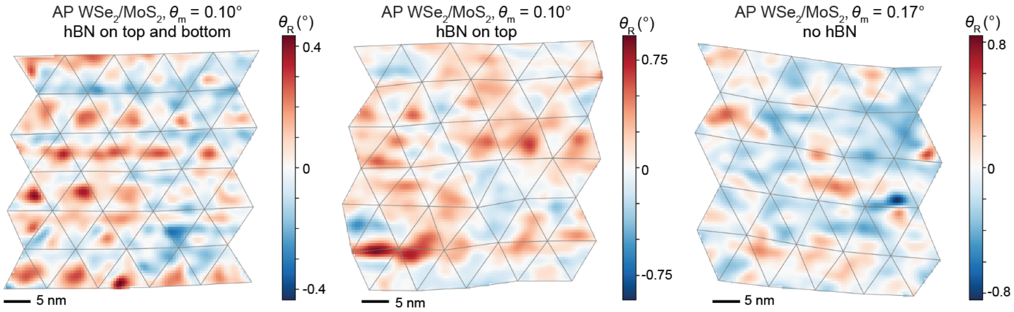

Supplementary Fig. 15. Effects of hBN on heterobilayer rotational reconstruction. Real-space maps of local rotations in AP WSe_2/MoS_2 ($\theta_m \approx 0.1$–$0.2°$) with varying extents of encapsulation.

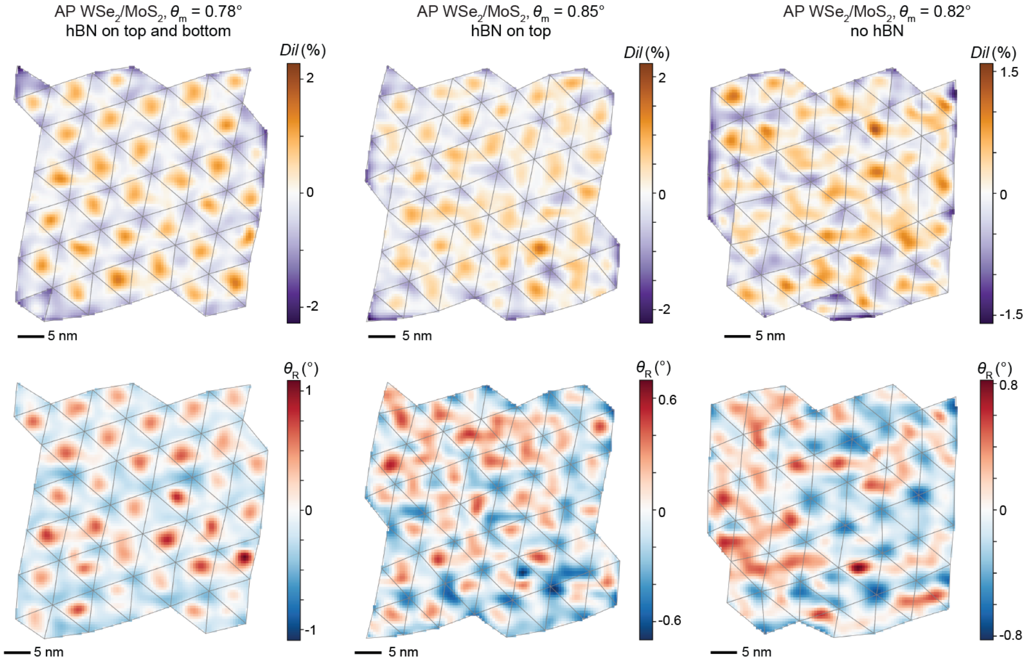

Supplementary Fig. 16. Effects of hBN on heterobilayer rotational and dilational reconstruction. Real-space maps of local dilations (top row) and rotations (bottom row) in AP WSe_2/MoS_2 ($\theta_m \approx 0.8°$) with varying extents of encapsulation.

10 Expected moiré lattice orientation for rotation calibration

Given an interlayer twist angle and lattice constant percent difference, we are able to compute the relative orientation between the lattice vectors associated with the moiré periodicity and those of the underlying atomic lattices. This was used to verify the rotational correction as described in the Methods section. Briefly, we first define $\mathbf{a}_1, \mathbf{a}_2$ as the average of the monolayer real space lattice vectors and orient $\mathbf{a}_1$ along the x-axis. Defining $\mathbf{A}_1, \mathbf{A}_2$ as the moiré lattice vectors, we can compute the angle φ between $\mathbf{a}_1$ and $\mathbf{A}_1$ as follows for a heterostructure with an interlayer twist of θ and lattice mismatch of $\delta = 1 - a_S/a_L$ (where a_S and a_L are the smaller and larger lattice constants respectively).

$$\varphi = \tan^{-1}\left(\frac{(\delta+1)^{-1}+1}{(\delta+1)^{-1}-1}\tan\left(\frac{\theta}{2}\right)\right) \tag{17}$$

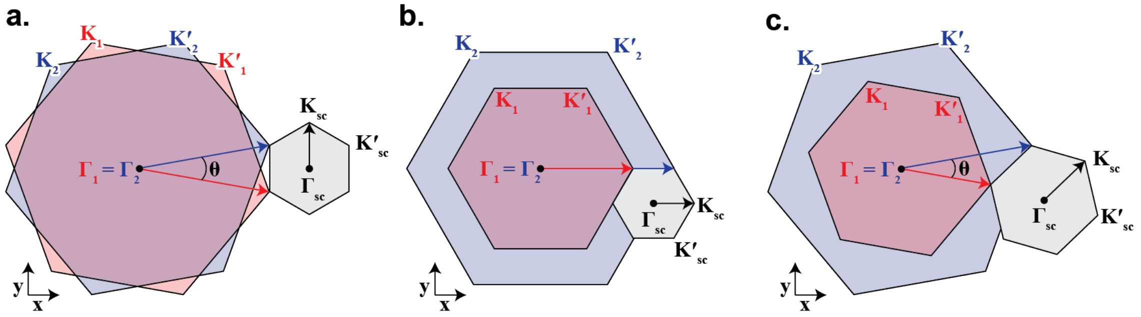

Supplementary Fig. 17. Orientation of moiré Brillouin zone for (**a**) a moiré homobilayer, (**b**) a lattice constant mismatch-driven moiré, and (**c**) a moiré superlattice resulting from both twist and lattice constant mismatch. We note that the reciprocal lattice vectors will be perpendicular to the K vectors shown for each atomic lattice (shown in red in blue for layers 1 and 2 respectively) and for the super-cell (depicted in grey).

This expression follows directly from a straightforward geometric analysis of the moiré Brillouin zones, presented in Supplementary Fig. 17, wherein the size and orientation of the

moiré reciprocal lattice vectors can be determined from the difference of the reciprocal lattice vectors associated with each atomic lattice. For a generic heterostructure, these atomic reciprocal lattice vectors are twisted with respect to each other and the vector associated with the smaller lattice is scaled by $1/(1-\delta)$.

11 Uncertainty in strain measurement

In order to estimate the percent uncertainty and detection limit of the reported strain calculations, we calculated the residuals between the raw disk intensities $I_{raw}(g_j)$ and the predicted $I_{fit}(g_j)$ following the displacement fitting procedure described in Supplementary Note 3. The mean and standard deviations of the residuals in the normalized intensities were on the order of -0.05 and 0.15 respectively, representing systematic bias and root mean squared error (RMSE) respectively. We note that this RMSE uncertainty in the intensities will reflect a larger displacement uncertainty in the $\mathbf{u} \approx 0$ domains in which the dependence of u on intensity is steeper. The small negative systematic bias in the intensity residuals is larger for samples with more background noise and reflects the fact that the high frequency noise not captured by the fitting function increases the average normalized intensity of the raw data. We believe the error from the fit procedure originates primarily from 1) experimental noise (carbon contamination, sample defects, and variation in tilt) causing a deviation from the expected intensity variation, 2) the validity of the approximations used in the fitting function derivation, and 3) the optimization procedure. We note that some of these effects may not be reflected in the obtained residuals and instead result in a good fit to a biased displacement value, which is difficult to quantify. Through collecting and averaging over strain values obtained at many different pixel locations, we are able to obtain a significantly lower standard error than through propagating the intensity uncertainties within a single pixel. The standard error measured is on the order of 0.1% dilation and a 0.1° rotation as seen in the presented strain values obtained across samples, which is associated with dilation and rotation detection limits roughly three times as large.

12 Supplementary References

1. Kim, K. *et al.* van der Waals heterostructures with high accuracy rotational alignment. *Nano Lett.* **16**, 1989–1995 (2016).

2. Maragkakis, G.M. *et al.* Imaging the crystal orientations of 2D transition metal dichalcogenides using polarization-resolved second-harmonic generation. *Opto-Electron. Adv.* **2**, 190026-1 (2019).

3. Vulović, M., Voortman, L.M., van Vliet, L.J. & Rieger, B. When to use the projection assumption and the weak-phase object approximation in phase contrast cryo-EM. *Ultramicroscopy* **136**, 61–66 (2014).

4. Kazmierczak, N.P. *et al.* Strain fields in twisted bilayer graphene. *Nat. Mater.* **20**, 956–963 (2021).

5. Latychevskaia, T. *et al.* Convergent beam electron holography for analysis of van der Waals heterostructures. *Proc. Natl. Acad. Sci.* **115**, 7473–7478 (2018).

6. Zachman, M.J. *et al.* Interferometric 4D-STEM for lattice distortion and interlayer spacing measurements of bilayer and trilayer 2D materials. *Small* **17**, 2100388 (2021).

7. Virtanen, P. *et al.* SciPy 1.0: fundamental algorithms for scientific computing in Python. *Nat. Methods* **17**, 261–272 (2020).

8. Van der Walt, S. *et al.* scikit-image: image processing in Python. *PeerJ* **2**, e453 (2014).

9. Hedengren, J.D., Shishavan, R.A., Powell, K.M. & Edgar, T.F. Nonlinear modeling, estimation, and predictive control in APMonitor. *Comput. Chem. Eng.* **70**, 133–148 (2014).

10. Beal, L., Hill, D., Martin, R. & Hedengren, J. GEKKO Optimization Suite. *Processes* **6**, 106 (2018).

11. Kerelsky, A. *et al.* Maximized electron interactions at the magic angle in twisted bilayer graphene. *Nature* **572**, 95–100 (2019).

12. Zeng, F., Zhang, W.-B. & Tang, B.-Y. Electronic structures and elastic properties of monolayer and bilayer transition metal dichalcogenides MX_2 (M= Mo, W; X= O, S, Se, Te): a comparative first-principles study. *Chin. Phys. B* **24**, 097103 (2015).

13. Yankowitz, M. *et al.* Emergence of superlattice Dirac points in graphene on hexagonal boron nitride. *Nat. Phys.* **8**, 382–386 (2012).

14. Carr, S. *et al.* Relaxation and domain formation in incommensurate two-dimensional heterostructures. *Phys. Rev. B* **98**, 224102 (2018).

15. Shabani, S. *et al.* Deep moiré potentials in twisted transition metal dichalcogenide bilayers *Nat. Phys.* **17**, 720–725 (2021).

16. Kresse, G. & Furthmüller, J. Efficient iterative schemes for ab initio total-energy calculations using a plane-wave basis set. *Phys. Rev. B* **54**, 11169–11186 (1996).

17. Peng, H., Yang, Z.-H., Perdew, J.P. & Sun, J. Versatile van der Waals Density Functional Based on a Meta-Generalized Gradient Approximation. *Phys. Rev. X* **6**, 041005 (2016).

18. Kresse, G. & Joubert, D. From ultrasoft pseudopotentials to the projector augmented-wave method *Phys. Rev. B* **59**, 1758–1775 (1999).

19. Mogensen, P.K. & Riseth, A.N. Optim: A mathematical optimization package for Julia. *Journal of Open Source Software* **3**, 615 (2018).

20. Enaldiev, V., Zólyomi, V., Yelgel, C., Magorrian, S. & Fal'ko, V. Stacking domains and dislocation networks in marginally twisted bilayers of transition metal dichalcogenides. *Phys. Rev. Lett.* **124**, 206101 (2020).

21. Li, H. *et al.* Imaging moiré flat bands in three-dimensional reconstructed WSe_2/WS_2 superlattices. *Nat. Mater.* **20**, 945–950 (2021).

22. Weston, A. *et al.* Atomic reconstruction in twisted bilayers of transition metal dichalcogenides. *Nat. Nanotechnol.* **15**, 592–597 (2020).
